# Knowledge, attitudes and practices regarding prevention of diabetic eye complications among people living with diabetes in Limpopo province of South Africa: A cross-sectional study

**DOI:** 10.4102/phcfm.v18i1.5411

**Published:** 2026-07-09

**Authors:** Khisimusi D. Maluleke, Saajida Mahomed

**Affiliations:** 1 School of Medicine, College of Health Sciences, University of KwaZulu-Natal, South Africa; 2 Department of Health, Sekororo District Hospital, Trichardtsdal, South Africa

**Keywords:** knowledge, attitudes, practice, patients, diabetic, retinopathy, complications, prevention

## Abstract

**Background:**

Diabetic retinopathy (DR) is a major cause of avoidable vision loss, yet public awareness is low, hindering early detection and treatment. Many people living with diabetes are unaware of their risk for DR and the need for screening.

**Aim:**

To assess the knowledge, attitudes and practices of people living with diabetes regarding DR prevention.

**Setting:**

Ten primary health care clinics in the Maruleng sub-district in Limpopo province of South Africa.

**Methods:**

A cross-sectional study utilised a self-reported questionnaire with yes/no questions to measure knowledge and practices, and attitudes were assessed with a 5-point Likert scale. Scores above the median for knowledge, attitudes and practices were indicative of good knowledge and practices and positive attitudes.

**Results:**

Almost two-thirds of participants (*n* = 255; 65.6%) were female. Approximately 268 participants (61.2%) knew that uncontrolled high blood sugar can lead to DR. The majority (*n* = 355; 86.1%) showed positive attitudes towards their eye health, while 216 (55.8%) had good practices towards DR prevention. Higher education and duration of diabetes were associated with good knowledge (adjusted odds ratio [AOR]: 2.2; 95% confidence interval [CI]: 1.3–3.9; *p* < 0.05) and (AOR: 1.9; 95% CI: 1.2–2.9; *p* < 0.05), respectively, while good knowledge about diabetic eye complications was associated with positive attitudes (AOR: 2.0; 95% CI: 1.3–3.1; *p* < 0.05). Patients diagnosed with diabetes mellitus for more than five years were more likely to attend regular eye exams (AOR: 2.3; 95% CI: 1.5–3.6; *p* < 0.05).

**Conclusion:**

Although participants had high median scores for knowledge and attitudes, important deficiencies were noted that could hinder effective DR screening.

**Contribution:**

These findings provide the baseline evidence on the need for ongoing patient education in primary care to enhance DR prevention.

## Introduction

Approximately one in nine adults aged 20 to 79 is living with either type 1 or 2 diabetes mellitus (DM), one of the chronic non-communicable diseases that threaten human health. Diabetes mellitus is a metabolic disease characterised by increased levels of blood glucose.^1^ In 2025, it was estimated that 830 million adults globally had some form of DM, with most living in low- and middle-income regions.^2^ Over 90% of adults affected have type 2 DM, and this has been largely attributed to unhealthy lifestyle choices, including consumption of a poor diet and physical inactivity.^3^

South Africa has more than 4 million people living with DM.^4^ Diabetes mellitus is a major public health problem, with many people at risk of developing microvascular complications.^2^ Diabetic retinopathy (DR) is one of the principal causes of vision impairment or loss that can affect up to 80% of people with type 1 DM after 15 years of being diagnosed, and over 60% of people with type 2 DM can develop DR within the first two decades.^5^ The prevalence of DR in South Africa varies among different studies, with some reporting rates from 34% to 42%.^6,7,8^ In Limpopo province of South Africa, a single-site study estimated the prevalence of DR at approximately 35%.^8^

While guidelines for DR screening and management are available, public awareness of DR is lacking compared with diseases such as HIV and tuberculosis. People with DM must be capacitated to manage their condition optimally to minimise the risk of vision loss because of DR. People living with DM should be aware of the need for regular eye examinations and strict control of blood glucose.^9^

The Health Belief Model posits that people’s understanding of health issues forms the foundation of their attitudes.^10^ In the context of DR prevention, this means people’s understanding of DM-related eye complications, treatment options and health behaviours can influence their feelings and beliefs about these topics, which then shape their actions and overall health practices, such as adhering to medication and attending regular eye examinations, and controlling blood sugar to prevent avoidable vision loss because of DR. Studies from urban and semi-urban healthcare settings in South Africa have reported knowledge gaps regarding diabetic eye complications among people with DM.^6,11,12^ However, no studies have been reported in rural areas.

This study aims to assess the knowledge, attitudes and practices (KAP) of people living with DM regarding DR prevention in the Maruleng sub-district, a rural community in the Mopani District of Limpopo province, South Africa.

## Research methods and design

### Study design

This was an observational cross-sectional study.

### Setting

The study was conducted in 10 of the 11 primary health care (PHC) clinics in the Maruleng sub-district, Mopani District of Limpopo province, South Africa. These clinics offer a range of PHC services, including the diagnosis and treatment of commonly occurring communicable and non-communicable diseases, family planning services and immunisations. These services are provided by nurses. A medical officer consults at the clinic weekly. For people with DM, the package of services includes medication collection, patient education and scheduled diabetic check-ups. These clinics do not provide any eye health services. People requiring eye care services are referred to the optometry clinic at Sekororo District Hospital. Maruleng is a largely rural sub-district with a population of approximately 128 137 people.^13^ The majority of people residing in this sub-district are of African descent, contributing 96% of the total population.^14^ The most spoken language is Sepedi (89.7%). The sub-district comprises 23 rural villages and one urban area.^14^

### Study population and sampling

The study included people with type 2 DM attending the 10 primary care clinics in Maruleng sub-district. The eleventh clinic that was excluded is a gateway clinic within Sekororo District Hospital, as patients attending this clinic tend to have easier access to services provided at the hospital than patients attending any of the other 10 primary care clinics. Patients under the age of 18 and those diagnosed with gestational diabetes were excluded. As the clinics did not have a list of patients expected per day, the principal investigator (PI) screened patients in the waiting area to see whether they were eligible to participate in the study. Thereafter, based on the headcount, every third patient at the busier clinics and every second patient at the quieter clinics were selected to spread sampling throughout the day. Data collection was done from Monday to Friday at each clinic.

### Sample size determination

The ideal sample size was calculated with Cochran’s single population formula, in which *Z* is the *z*-score representing the desired confidence level (here it is equal to 1.96 for a 95% confidence interval [CI]), *p* is the estimated population proportion, which was set at 0.5 when unknown, and *e* is the margin of error expressed as a 0.05 decimal.^15^ Thus, the required sample size (*n*) of this study is 384 (Equation 1):


$$n=\frac{Z^{2}p(1-p)}{e^{2}}=\frac{(1.96{)}^{2}0.50(1-0.50)}{(0.05{)}^{2}}=384$$ [Eqn 1]

The sample size was split across the 10 clinics proportional to the headcount at each clinic.

### Data collection tool and procedure

The data were gathered utilising a self-reported structured questionnaire with four sections: Sociodemographic characteristics and KAP. The questionnaire was adapted from a previously validated research instrument to measure KAP regarding DR among peoples with DM.^16^ A context-specific adaptation of this questionnaire included removing some demographic items, rephrasing knowledge items and adding new practice and attitude items. Face validity was achieved through expert review. A pilot study was conducted among 10 patients at the Tzaneen primary care clinic in a different sub-district to confirm content and face validity. The Pearson’s correlation score was 1.00 for all items. We calculated Cronbach’s alpha to assess the questionnaire’s internal consistency. The results showed the alpha scores of 0.8 for knowledge, 0.6 for attitudes and 0.7 for practices, indicating a reliable questionnaire. The questionnaire was created in English and translated into Sepedi, the local language spoken in the area. The Sepedi version was then back-translated to English to ensure its original meaning was preserved.

Knowledge and practices were assessed with binary responses (yes/no), and attitudes were assessed using a 5-point Likert scale (strongly disagree to strongly agree). The primary research data were collected through a paper-based survey conducted over 3 months during various clinic visits in 2024. Prior to the survey, participants were instructed to respond honestly to each question after providing their consent. For individuals who were illiterate, informed consent was read out to them, and an ink stamp was used to obtain their right thumbprints as a form of consent, and the PI read the questions and recorded the patients’ responses. Each participant received a unique identification number, and to maintain confidentiality, no personal information was collected from them.

### Data analysis

The data were initially entered in a Microsoft Excel spreadsheet (Microsoft 365, Microsoft Corp, Redmond, Washington, United States) and exported to Statistics and Data Analysis (Stata) software edition 15 (StataCorp, College Station, Texas, United States) for analysis. Knowledge and practice responses were assigned 1 point for correct answers and 0 points for incorrect answers, and a total was calculated for each section per patient. Similarly, for attitude statements, favourable responses were assigned 1 point, and neutral or unfavourable responses were assigned 0 points. We performed the Shapiro–Wilk test to assess the normality of the data distribution normality. After confirming non-normal data distributions, the median and interquartile range were used for continuous variables with skewed distributions. Because of the asymmetrical distribution of the KAP scores, the median score, representing the 50th percentile, was used as a threshold to categorise KAP as good or positive (equal to or above the median) and poor or negative (below the median). Percentages and frequencies were used to display categorical data. We used the chi-square (χ^2^) test with a significance level of *p* < 0.05 to evaluate the association between demographic variables and KAP scores. Bivariate and multivariate logistic regression analyses were utilised to identify the determinants of KAP; odds ratio (OR) was reported with 95% CI, which aided interpretation of the findings. A stepwise selection strategy was used to select important variables for the analysis model. A statistically significant adjusted odds ratio (AOR) was defined as *p* < 0.05 in this analysis. Variables that lacked statistical significance in the bivariate analysis but had been found in previous studies to influence patients’ KAP were also included in the multivariate analysis. Highly related variables because of collinearity were excluded from multivariate analysis.

### Ethical considerations

Ethical clearance to conduct this study was obtained from the Biomedical Research and Ethics Committee (BREC), University of KwaZulu-Natal (No. BREC/0000631172023). The World Medical Association’s Declaration of Helsinki, which provides a fundamental framework for ethical research, was upheld by this study, which complied with all ethical requirements for medical-related studies involving human subjects. The National Department of Health, the Provincial Health Research Committee under the Limpopo Department of Health (LP_202404_003) and the Mopani District authorised the gatekeeper. Participants’ information was kept confidential and not shared with third parties without permission, ensuring protection from unauthorised access or loss. Every participant gave their informed written consent.

## Results

### Sociodemographic profile

A total of 389 people living with diabetes voluntarily participated in the study. Table 1 presents a summary of the sociodemographic characteristics of the participants. The median age was 62 years (interquartile range [IQR]: 55–69 years), and the median duration since diagnosis of DM was 7 years (IQR: 2–14 years). There was no significant difference in the median age between males and females (results not shown). Most participants were female (*n* = 255; 65.6%). More than half of the participants (*n* = 231; 59.4%) were pensioners, and 103 (26.5%) had no education.

**TABLE 1:** Sociodemographic characteristics of people living with diabetes in Maruleng sub-district, Limpopo province, 2024 (*N* = 389).

Variables	Categories	Number	%
Age groups (years)	≤ 62	204	52.4
	> 62	185	47.6
Gender	Female	255	65.6
	Male	134	34.4
Level of education	None	103	26.5
	Primary	128	32.9
	Secondary	116	29.8
	Tertiary	42	10.8
Employment status	Employed	51	13.1
	Unemployed	107	27.5
	Pensioner	231	59.4
Smoking status	Smoker	37	9.5
	Not smoker	352	90.5
Drinking alcohol	Drinking	34	8.7
	Not drinking	355	91.3
Time since diabetes diagnosis	5 years or less	152	39.1
	More than 5 years	237	60.9

### Knowledge about diabetic eye complications

The median knowledge score was 80% (IQR: 40–100). Approximately 268 (61.2%) participants had a score above the median, indicating good knowledge of diabetic eye complications. More than two-thirds of participants (*n* = 277, 71.2%) reported that eye problems because of diabetes can be treated, and just over half of the participants (*n* = 233, 59.9%) were aware that they should undergo an eye examination (Table 2).

**TABLE 2:** Patients’ knowledge on diabetic eye complications in Maruleng sub-district, Limpopo province, 2024.

Items	Knowledgeable			
	Yes		No	
	*n*	%	*n*	%
1. Have you heard about eye complications because of diabetes?	322	82.8	67	17.2
2. Should patients diagnosed with diabetes have an eye examination?	233	59.9	156	40.1
3. Can diabetic eye complications cause blindness?	280	72.0	109	28.0
4. If my blood sugar is not well controlled, I am at risk of eye problems.	260	66.8	129	33.2
5. Eye problems because of diabetes can be treated.	277	71.2	112	28.8

### Attitudes of patients towards prevention

The median attitude score was 85.7% (IQR: 71.4–100), with the majority of patients (*n* = 335, 86.1%) having a score above the median, indicating a positive attitude towards diabetic eye complications. More than three-quarters (*n* = 301, 77.4%) of the patients disagreed that there are more important issues to worry about than the eye-related problems caused by DM. Eighty-four patients (21.6%) agreed that they do not go for an eye examination because no one told them, and 44 (11.3%) agreed that they do not go for an eye examination because the hospital was far from where they live (Table 3).

**TABLE 3:** Patients’ attitudes towards prevention of diabetic eye complications in Maruleng sub-district, Limpopo province, 2024.

Items	Attitude levels									
	Strongly disagree		Moderately disagree		Neutral		Moderately agree		Strongly agree	
	*n*	%	*n*	%	*n*	%	*n*	%	*n*	%
I am afraid to ask doctors or nurses questions about diabetic eye complications	239	61.4	75	19.3	22	5.7	33	8.5	20	5.1
There are more important health issues to worry about than eye problems	205	52.7	96	24.6	38	9.8	26	6.7	24	6.2
I do not go for an eye examination because nobody told me to	182	46.8	97	24.9	26	6.7	43	11.1	41	10.5
I do not go for an eye examination because the hospital is too far away from my place	233	59.9	92	23.7	20	5.1	23	5.9	21	5.4
Listening to health talks at the community clinic or hospital is a waste of time for patients with diabetes	245	62.9	80	20.6	16	4.1	24	6.2	24	6.2

### Practice of people living with diabetes towards prevention

The median practice score was 42.9% (IQR: 28.6–71.4). Only 216 (55.8%) of the patients scored above the median, indicating good practices towards DR prevention. Of the 115 people living with DM who missed their treatment, 56 (48.7%) skipped a dose for just 1 day. Of the 183 patients (47%) who reported having visual problems, 148 (80.9%) informed their doctor or nurse about the issue; 90 (23.1%) of these patients were subsequently referred to the nearest optometrist for an eye examination and further management, 76 (19.5%) were treated with spectacles and eye drops by optometrist and 16 (4.1%) reported that nothing was done to address their visual problems. More than 50% of patients had never undergone an eye examination, and 110 (28.3%) reported visiting an optometrist without referral (Table 4).

**TABLE 4:** Patients’ practices of preventing diabetic eye complications in Maruleng sub-district, Limpopo province, 2024.

Items	Responses	*n*	%
1. Have you ever missed a dose of your diabetes medication?	Yes	115	29.6
	No	274	70.4
2. Have you ever had an eye examination?	Yes	179	46.0
	No	210	54.0
3. Have you ever had any visual problems?	Yes	183	47.0
	No	206	53.0
4. If yes to question 3, have you ever told the doctor or nurse about it	Yes	148	38.1
	No	241	61.9
5. Have you ever told the doctor or nurse about diabetic eye complications?	Yes	148	38.0
	No	241	62.0

### Factors associated with patients’ knowledge of diabetic eye complications

Patients with educational background including primary and above (secondary and tertiary educations) were twice more likely to know about diabetic eye complications compared to those without any education, with an AOR of 1.9; 95% CIs: 1.1–3.3; *p* < 0.05 and AOR: 2.2; 95% CI: 1.3–3.9; *p* < 0.05, respectively. Additionally, patients diagnosed with DM for more than 5 years were more likely to have good knowledge than those diagnosed less than 5 years ago (AOR: 1.9; 95% CI: 1.1–2.9; *p* < 0.05) (Table 5).

**TABLE 5:** Factors associated with patients’ knowledge of diabetic eye complications.

Variables	Categories	Good		Poor		Crude odds ratio			Adjusted odds ratio		
		*n*	%	*n*	%	OR	95% CI	*p*-value	OR	95% CI	*p*-value
Age (years)	≤ 62	123	31.6	81	20.8	Reference	1	-	Reference	1	-
	> 62	115	29.6	70	18.0	1.1	0.7–1.6	0.706	†	-	**
Gender	Males	88	22.6	46	11.8	Reference	1	-	Reference	1	-
	Females	150	38.6	105	27.0	1.3	0.9–2.1	0.188	1.2	0.8–1.9	0.349
Level of education	None	50	12.9	50	12.9	Reference	1	-	Reference	1	-
	Primary	88	22.6	40	10.3	1.6	1.0–2.7	0.055	1.9	1.1–3.2	**0.021**
	Secondary and Tertiary	100	25.7	61	15.7	2.2	1.3–3.8	**0.004**	2.2	1.3–3.9	**0.007**
Time since diagnosed (years)	< 5	82	21.1	70	18.0	Reference	1	-	Reference	1	-
	> 5	156	40.1	81	20.8	1.6	1.1–2.5	**0.019**	1.9	1.2–2.9	**0.005**

Note: bold values are significant level *p* < 0.05.

*n*, number of observations; OR, odds ratio; CI, confidence intervals.

** , Excluded from multivariate analysis because of collinearity;

† , Excluded from multivariate analysis because of collinearity.

### Factors associated with patients’ attitudes towards prevention

In multivariate analysis, patients with good knowledge of diabetic eye complications were twice more likely to have positive attitudes towards the prevention of diabetic eye complications than those with poor knowledge (AOR: 2.0; 95% CI: 1.3–3.1; *p* < 0.005) (Table 6).

**TABLE 6:** Factors associated with patients’ attitudes towards prevention of diabetic eye complications.

Variables	Categories	Positive		Negative		Crude odds ratio			Adjusted odds ratio		
		*n*	%	*n*	%	OR	95% CI	*p*-value	OR	95% CI	*p*-value
Age (years)	≤ 62	122	31.4	82	21.1	Reference	1	-	Reference	1	-
	> 62	118	30.3	67	17.2	1.2	0.8–1.8	0.420	†	-	**
Gender	Males	89	22.9	45	11.6	Reference	1	-	Reference	1	-
	Females	151	38.8	104	26.7	1.3	0.9–2.1	0.166	1.4	0.9–2.2	0.140
Level of education	None	66	17.0	34	8.7	Reference	1	-	Reference	1	-
	Primary	81	20.8	47	12.1	0.7	0.4–1.2	-	0.7	0.4–1.3	0.249
	Secondary and Tertiary	93	23.9	68	17.5	0.9	0.5–1.5	-	0.6	0.4–1.1	0.079
Time since diagnosed (years)	≤ 5	89	22.9	63	16.2	Reference	1	-	Reference	1	-
	> 5	151	38.8	86	22.1	1.2	0.8–1.9	0.307	1.1	0.7–1.7	0.637
Knowledge	Poor	77	19.8	74	19.0	Reference	1	-	Reference	1	-
	Good	163	41.9	75	19.3	2.1	1.4–3.2	**0.001**	2.0	1.3–3.1	**0.001**

Note: bold values are significant level *p* < 0.005.

** , Excluded from multivariate analysis because of collinearity;

† , Excluded from multivariate analysis because of collinearity.

*n*, number of observations; OR, odds ratio; CI, confidence intervals.

### Factors associated with patients’ practice of prevention

Patients diagnosed with DM more than 5 years were twice more likely to attend regular eye examinations than those diagnosed less than 5 years ago (AOR: 2.3; 95% CI: 1.5–3.6; *p* < 0.005) (Table 7).

**TABLE 7:** Factors associated with patients’ practice of preventing diabetic eye complications.

Variables	Categories	Good		Bad		Crude odds ratio			Adjusted odds ratio		
		*n*	%	*n*	%	OR	95% CI	*p*-value	OR	95% CI	*p*-value
Age (years)	≤ 62	104	26.7	100	25.7	Reference	1	-	Reference	1	-
	> 62	109	28.0	76	19.5	1.4	0.9–2.1	0.117	†	-	**
Gender	Males	65	16.7	69	17.7	Reference	1		Reference	1	-
	Females	148	38.0	107	27.5	0.7	0.4–1.0	0.073	0.7	0.5–1.1	0.111
Level of education	None	59	15.2	41	10.5	Reference	1		Reference	1	-
	Primary	69	17.7	59	15.2	0.8	0.5–1.3	0.328	0.9	0.5–1.5	0.588
	Secondary and Tertiary	85	21.9	76	19.5	0.8	0.5–1.4	0.442	1.0	0.6–1.7	0.960
Time since diagnosed (years)	≤ 5	67	17.2	85	21.9	Reference	1		Reference	1	-
	> 5	146	37.5	91	23.4	1.1	1.0–1.1	**< 0.001**	2.3	1.5–3.6	**< 0.001**
Knowledge	Poor	79	20.3	72	18.5	Reference	1	-	Reference	-	-
	Good	134	34.4	104	26.7	1.2	0.8–1.8	0.442	1.1	0.7–1.7	0.686
Attitudes	Negative	80	20.6	69	17.7	Reference	1	-	Reference	1	-
	Positive	133	34.2	107	27.5	1.1	0.7–1.6	0.740	1.0	0.7–1.6	0.879

Note: bold values are significant level *p* < 0.001.

** , Excluded from multivariate analysis because of collinearity;

† , Excluded from multivariate analysis because of collinearity.

*n*, number of observations; OR, odds ratio; CIs, confidence intervals.

## Discussion

This is the first published research specifically focusing on all three aspects of the KAP model among people with DM in a rural community in South Africa regarding diabetic eye complications. Almost two-thirds of the participants (65.6%) were female, consistent with findings from the South African Demographic Health Survey, which reported that 24.7% of females were diabetic compared to 17.2% of males.^17^ A previous study conducted among people living with diabetes in South Africa also reported a high percentage (62%) of female patients.^18^ The increased proportion of females aligns with findings from a sub-Saharan systematic review showing that females were more likely to seek healthcare than males.^19^

Although 61.2% of patients had good knowledge, there were deficiencies, especially regarding specific aspects of diabetic eye complications (DR), including screening frequency, available treatments and the fact that early stages of DR often have no symptoms. Knowledge gaps often lead to poor adherence to recommended eye care practices, such as regular screenings, despite awareness of the risks. A lack of understanding about diabetic eye issues hinders screening programmes for DR, increasing the risk of preventable sight-threatening complications.^20,21,22^

Patients’ educational background was significantly associated with good knowledge. Higher levels of formal education, including secondary or tertiary education, are consistently linked to better knowledge of diabetic eye complications, as these patients can read about DR and its associated risk factors and tend to attend recommended eye examinations and seek timely treatment, which is crucial for early detection and prevention.^16,23^ Patients with lower literacy levels are less likely to understand complex health information. Duration of diabetes was also associated with good knowledge. This association is likely explained by these patients having had greater opportunities to receive education about diabetic eye complications, and that they would be at greater risk of DR compared to patients with a shorter duration of diabetes.^24,25,26^

In this study, the majority of people living with diabetes exhibited positive attitudes towards diabetic eye complications, in keeping with earlier studies from Sudan and Oman.^27,28^ Patients with good knowledge were more likely to have positive attitudes, consistent with the premise of the KAP framework that acquiring knowledge builds attitude.^29^ Fostering positive attitudes among people living with diabetes is crucial for promoting health-seeking behaviours and acceptance of vital information about preventing diabetic eye complications.^20^ In turn, a positive attitude encourages better practices for managing and preventing diabetic eye complications.^30^

Despite the majority of patients having good knowledge and positive attitudes towards DR, their preventative practices for eye complications were poor, as indicated by a median practice score of 42.9%. Practices for preventing diabetic eye complications can be influenced by a combination of patient-related, provider-related and systemic barriers.^31,32,33^ Patients often overlook the critical importance of regular dilated eye exams, especially when they do not experience any visual symptoms.^34^ Additionally, patients may fail to adhere to treatment plans for managing diabetes, which is the key risk factor for having DR. Many patients mistakenly ignore early symptoms of DR, considering them a “normal” part of living with diabetes.^32^ Moreover, fatalistic beliefs about diabetic complications, low motivation and anxiety about receiving a negative diagnosis can lead to avoidance of screening tests.^35^

Systemic barriers, such as financial constraints, overcrowded clinics, long waiting times, inadequate infrastructure leading to poor healthcare access issues and poor coordination, in DR screening programme for patients with DM on treatment, further exacerbate the problem of delayed screening and avoidance of utilising the essential eye care services.^31,36^ The majority of affected people were unemployed, earned little or were pensioners, which can result in limited transportation options – especially for those living in rural areas. This lack of transportation may cause patients to prioritise other needs over their eye care, leading to delays in DR screening.^37^ Socioeconomic factors, including lower educational attainment, poor literacy and social deprivation, are also associated with reduced adherence to eye screening guidelines.^38^

People diagnosed with DM for more than 5 years were more likely to utilise eye care services and have regular eye check-up practices than those diagnosed for a shorter duration. This may be because of a higher incidence of ocular problems over time, prompting them to seek medical attention, or to repeated interactions with healthcare providers that reinforce the need for screening. This finding is consistent with a review of eye care service utilisation and associated factors among people living with DM in Africa.^36^

This study has some limitations. Although the questionnaire was back-translated and piloted, translating Likert scale questions was a complex process. There are nuances in the language and cultural differences in response styles that may have led to information bias. Furthermore, response bias is a possibility when self-reported practices are used. Systematic sampling should have resulted in a random and reasonably representative sample. This temporal variety helps mitigate bias by including diverse participants. The results cannot be applied to all South African health districts, especially urban ones, as the research was restricted to a single sub-district in Limpopo province.

### Recommendations

The authors suggest adopting a system-level approach that uses mobile units to reach patients in rural communities or underserved areas. This strategy should involve a multidisciplinary team, including eye care professionals such as optometrists, as part of the diabetes management team. The use of mobile fundus cameras at the primary care level could be a highly effective strategy for accelerating retinal screening among people living with diabetes. Training PHC nurses to capture high-quality retinal images can bring screening directly to underserved or rural areas, thereby addressing barriers such as transportation and long appointment wait times. By integrating eye care earlier in the treatment process, we can ensure patients receive consistent, specialised care that addresses knowledge gaps, fosters positive attitudes and promotes best practices related to DR. Healthcare professionals can empower patients to take proactive measures to prevent vision loss and maintain their eye health.

## Conclusion

People living with DM in this rural area of South Africa reported good knowledge and positive attitudes towards preventing diabetic eye complications. However, patients reported poor practices with regard to recommended eye examinations and are thus at risk for diabetic eye complications because of a combination of suboptimal systemic control of related conditions, inconsistent screening adherence, healthcare system barriers and non-modifiable risk factors like genetics and disease duration. We have concluded that there is a significant deficiency in the uptake of regular eye examinations. The results further underscore the need to raise awareness of diabetic eye complications and improve patient education in the PHC setting. Such initiatives are essential for fostering positive attitudes towards DR prevention and promoting good practices, such as attending regular eye examinations, ultimately aiming to prevent vision impairment or loss associated with DR.
